# Dual Polarization of Ni Sites at VO_x_−Ni_3_N Interface Boosts Ethanol Oxidation Reaction

**DOI:** 10.1002/advs.202407473

**Published:** 2024-09-03

**Authors:** Min Zhou, Binrong Jin, Weijie Kong, Anjie Chen, Yuhe Chen, Xiuyun Zhang, Fei Lu, Xi Wang, Xianghua Zeng

**Affiliations:** ^1^ College of Physical Science and Technology Yangzhou University Yangzhou 225002 P. R. China; ^2^ Microelectronics Industry Research Institute Yangzhou University Yangzhou 225002 P. R. China; ^3^ Department of Physics, School of Physical Science and Engineering Beijing Jiaotong University Beijing 100044 P. R. China

**Keywords:** dual polarization, ethanol oxidation reaction, interface engineering, nickel nitride, vanadium oxide

## Abstract

Substituting thermodynamically favorable ethanol oxidation reaction (EOR) for oxygen evolution reaction (OER) engenders high‐efficiency hydrogen production and generates high value‐added products as well. However, the main obstacles have been the low activity and the absence of an explicit catalytic mechanism. Herein, a heterostructure composed of amorphous vanadium oxide and crystalline nickel nitride (VO_x_−Ni_3_N) is developed. The heterostructure immensely boosts the EOR process, achieving the current density of 50 mA cm^−2^ at the low potential of 1.38 V versus reversible hydrogen electrode (RHE), far surpassing the sluggish OER (1.65 V vs RHE). Electrochemical impedance spectroscopy indicates that the as‐fabricated heterostructure can promote the adsorption of OH^−^ and the generation of the reactive species (O^*^). Theoretical calculations further outline the dual polarization of the Ni site at the interface, specifically the asymmetric charge redistribution (interfacial polarization) and in‐plane polarization. Consequently, the dual polarization modulates the d‐band center, which in turn regulates the adsorption/desorption strength of key reaction intermediates, thereby facilitating the entire EOR process. Moreover, a VO_x_−Ni_3_N‐based electrolyzer, coupling hydrogen evolution reaction (HER) and EOR, attains 50 mA cm^−2^ at a low cell voltage of ≈1.5 V. This work thus paves the way for creating dual polarization through interface engineering toward broad catalysis.

## Graphical abstract

A meticulously engineered heterostructure has been developed, integrating amorphous vanadium oxide with crystalline nickel nitride (VO_x_−Ni_3_N), featuring a well‐defined Ni─O─V interface. This dual polarization at the Ni site within the interface significantly enhances the efficiency of the ethanol oxidation reaction (EOR). Furthermore, an electrolyzer configured for EOR‐coupled hydrogen production has been constructed and successfully powered by a commercial dry battery.

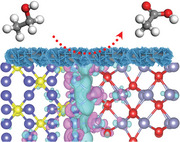

## 1 Introduction

With increasing energy and environmental concerns, electrocatalytic water splitting for hydrogen production has become a prominent topic in recent years.^[^ ^1^, ^2^, ^3^, ^4^ ^]^ Nevertheless, the sluggish kinetics of the anodic oxygen evolution reaction (OER) limits the efficiency of the overall water splitting.^[^ ^5^, ^6^, ^7^, ^8^ ^]^ One promising approach is utilizing the thermodynamically more favorable ethanol oxidation reaction (EOR) in place of the OER, which shows great potential as an alternative to conventional electrolytic water splitting for hydrogen production.^[^ ^9^, ^10^ ^]^

In previous studies, transition metal‐based nanomaterials have been proven to be efficient for EOR, particularly nickel and cobalt‐based compounds, such as NiO,^[^ ^11^ ^]^ Ni(OH)_2_,^[^ ^12^ ^]^ Co_3_O_4_,^[^ ^13^, ^14^ ^]^ Co_3_S_4_,^[^ ^15^ ^]^ and so on. The catalytic activity of the mentioned catalyst is primarily attributed to the high‐valence metals. However, a higher applied potential (>1.35 V vs RHE) is required to achieve the specific valence state, which is far surpassing the EOR thermodynamic potential (<0.5 V vs RHE).^[^ ^16^, ^17^ ^]^ As a result, the higher activation potential of transition metal‐based chalcogenides limits the efficiency in EOR application. Therefore, it is crucial to explore efficient EOR electrocatalysts with lower activation potential and further clarify their reaction mechanisms.

Transition metal nitrides (TMNs), due to their various electrocatalytic properties and excellent corrosion resistance, are widely used in various catalysis.^[^ ^18^, ^19^ ^]^ However, according to the Sabatier principle, the monoactive species could not favor the adsorption and activation of the involved intermediates. Thus, it remains challenging to regulate multi‐step proton‐coupled electron transfer.^[^ ^20^, ^21^ ^]^ Given this, Song et al. synthesized defective carbon–CoP nanoparticle hybrids and applied them in oxygen catalysis.^[^ ^22^ ^]^ It revealed that the interfacial charge polarization, with the electrons gathering at the defective carbon surface, facilitates the activation of various oxygen‐involved intermediates. Ma's group reported that the built‐in interfacial polarization between BP nanoflakes and Nb_2_C nanosheets triggered high‐efficiency electrochemical nitrate reduction.^[^ ^23^ ^]^ Theoretical calculations demonstrated that the enhanced NO_3_^−^RR performance of the BP/Nb_2_C composite is attributed to the presence of polarization effects between BP nanoflakes and Nb_2_C nanosheets, leading to a high electron distribution at the interface of the BP/Nb_2_C composite. Therefore, interface engineering in heterostructures, especially the interfacial polarization, is an effective means to maneuver the active sites in close proximity to synergistically improve the intrinsic activity of catalysts and thus enhance the catalytic performance.

Herein, we reported on the synthesis of a VO_x_−Ni_3_N heterostructure, specifically integrating amorphous VO_x_ with crystalline Ni_3_N. Dual polarization, including the in‐plane polarization and interfacial polarization between the hybrid phase, is demonstrated by detailed characterization. As a result, the VO_x_−Ni_3_N delivers superior EOR and HER catalytic activity and durability in alkaline ethanol media. By assembling an ethanol electrolyzer using VO_x_−Ni_3_N as a bifunctional electrocatalyst, a current density of 50 mA cm^−2^ could be achieved at a voltage of ≈1.5 V, comparable to the charge from a commercial dry battery. Consequently, high‐value‐added acetic acid could be produced at a cost‐effective route. Moreover, in‐situ electrochemical impedance spectroscopy and theoretical calculation analyses reveal the catalytic mechanism. The asymmetric charge distribution induced by dual polarization synergistically facilitates the coupling of O_ads_ and ethanol at the VO_x_−Ni_3_N interface, thus boosting the catalytic performance. Therefore, creating dual−polarization through interface engineering could effectively promote multi‐step proton‐coupled electron transfer during heterogeneous catalysis beyond the Sabatier principle.

## 2 Results and Discussion

### 2.1 Material Characterization

**Figure 1:**
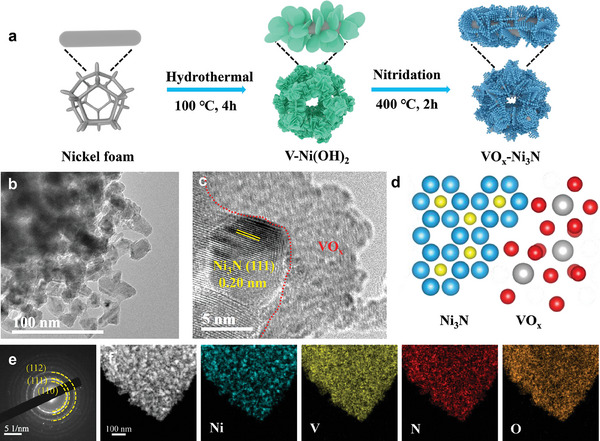
Structural characterization of the materials: a) Schematic of catalyst synthesis. TEM b) and HRTEM c) images of the VO_x_−Ni_3_N. d) Schematic atomic structure of VO_x_−Ni_3_N. e) Selected area electron diffractogram (SAED) and f) Scanning transmission electron microscopy (STEM) image and the corresponding elemental mapping.

The VO_x_−Ni_3_N heterostructure was synthesized by sequential hydrothermal reaction and annealing process, as schematically illustrated in **Figure** **1**a. The V doped nickel layered double hydroxide (Ni–V LDH) precursor was prepared through a hydrothermal approach. Then, VO_x_−Ni_3_N heterostructure was obtained by annealing the precursor at 400 °C in a NH_3_ atmosphere. The crystalline Ni_3_N phase in VO_x_−Ni_3_N heterostructure, indexed to PDF#10–0280, is confirmed by the X‐ray diffraction (XRD) pattern,^[^ ^23^ ^]^ while no other diffraction signals are observed. It suggests that vanadium might present in an amorphous state since no diffraction peak attributed to vanadium‐related structure is observed (Figure S1, Supporting Information).

The morphology of the obtained VO_x_−Ni_3_N heterostructure was characterized by field‐emission scanning electron microscopy (FESEM) and transmission electron microscopy (TEM). As shown in Figure S2 (Supporting Information), the Ni–V LDH is depicted with nanosheets that are well‐aligned and self‐assembled into a spherical structure. The nanosheet shape is inherited with rough and porous surfaces for VO_x_−Ni_3_N heterostructure after annealing. From the typical TEM image (Figure 1b), it is observed that the nanosheets are composed of interconnected nanoparticles, which might be caused by the phase transformation upon the annealing process. The lattice spacing of 0.20 nm corresponds to the (111) crystal plane of Ni_3_N (Figure 1c), consistent with selected area electron diffractogram (SAED) signatures in Figure 1e, as the schematic diagram shown in Figure 1d. Moreover, the amorphous phase at the edge of crystalline Ni_3_N can be identified. Given the elemental mapping profiles in Figure 1f, the margin of the entire heterostructure is composed by vanadium and oxygen. Thus, the observed amorphous phase can be attributed to VO_x_, which keeps a non‐crystalline state due to the relatively lower annealing temperature. Therefore, amorphous vanadium oxide coupled with crystalline nickel nitride can be demonstrated for the obtained VO_x_−Ni_3_N heterostructure.

**Figure 2:**
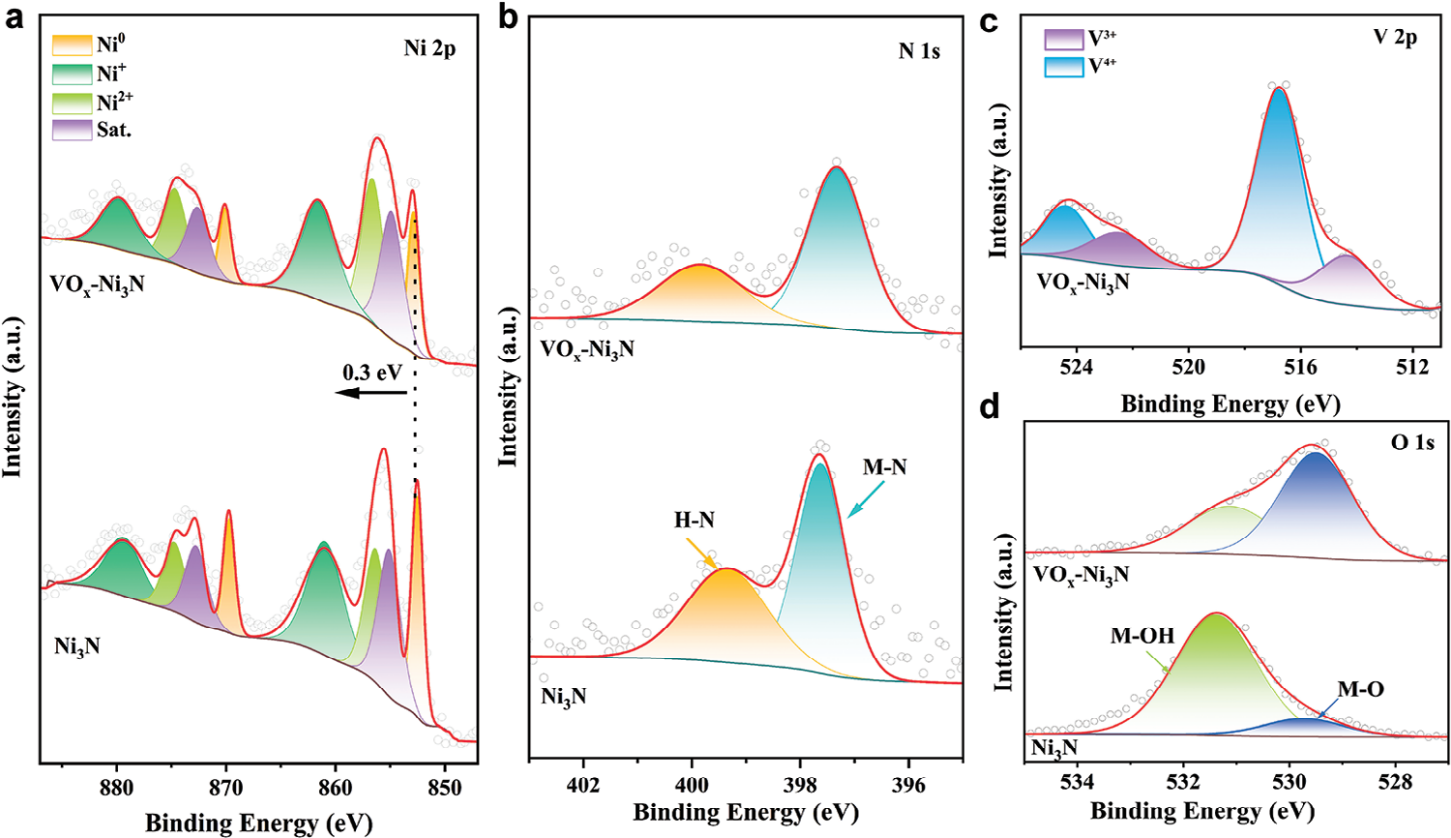
XPS spectra of Ni_3_N and VO_x_−Ni_3_N: Ni 2p a), N 1s (b). c) V 2p XPS spectrum of VO_x_−Ni_3_N and O 1s spectra of Ni_3_N and VO_x_–Ni_3_N (d).

X−ray photoelectron spectroscopy (XPS) was employed to investigate the electronic structure of the VO_x_−Ni_3_N heterostructure. In the Ni 2p spectra (**Figure** **2**a), the peak pairs 852.2/869.5, 855.3/872.8, 856.1/873.8, and 860.6/878.9 eV can be well assigned to the binding energies of Ni─Ni, Ni─N, and Ni─O, and Sat. peaks.^[^ ^24^, ^25^ ^]^ Compared with that of the bare Ni_3_N, a positive shift of 0.3 eV in binding energies is detected, indicating the enhanced electron delocalization of the Ni_3_N after V is introduced. Meanwhile, Ni─O signals increase accompanied by a decrease in Ni─N intensity. The lifted Ni─O signature is potentially attributed to Ni_3_N bonded VO_x_ species, different from surface oxidation. The migration of delocalized electrons from Ni_3_N to VO_x_ at the interface is indicative of interfacial polarization. Then, a novel Ni─O─V interface is deduced within the VO_x_−Ni_3_N heterostructure.^[^ ^26^ ^]^ As a result, a prominent charge redistribution occurs within the heterostructure, especially the Ni─O─V interface.

In the N 1s spectrum (Figure 2b) the peaks of 397.6 and 399.4 eV belong to the Ni─N and N─H bonds, respectively. Notably, the Ni─N bond undergoes a negative shift of 0.3 eV, indicating a weak Ni─N interaction in VO_x_−Ni_3_N.^[^ ^27^ ^]^ This is quite consistent with the formation of the Ni─O─V interface, whilst diminishing the Ni─N species. Then, the in‐plane polarization of nickel sites within the VO_x_‐Ni_3_N heterostructure is evident from the emerging Ni─N species relative to the metallic nickel, especially the nickel sites at the surface. For vanadium, the hybrid valence of both 3+ and 4+ is confirmed in Figure 2c, as two sets of peaks are shown in the V 2p_1/2_ range. The prominent peak at 513.7 eV indicates the dominated V_2_O_3_ species in the amorphous VO_x_. The higher binding energy related to V^4+^ species might be attributed to the formed Ni─O─V interface, particularly the charge redistribution within it.^[^ ^28^, ^29^ ^]^ The shift of the corresponding M─O binding energy in O1s spectra also confirms the mixed metal–oxygen species (Figure 2d), consistent with the emerging Ni─O─V interface in the VO_x_−Ni_3_N heterostructure. Therefore, it can be concluded that introducing V into Ni_3_N encourages the formation of the Ni─O─V interface in the VO_x_−Ni_3_N heterostructure.

**Figure 3:**
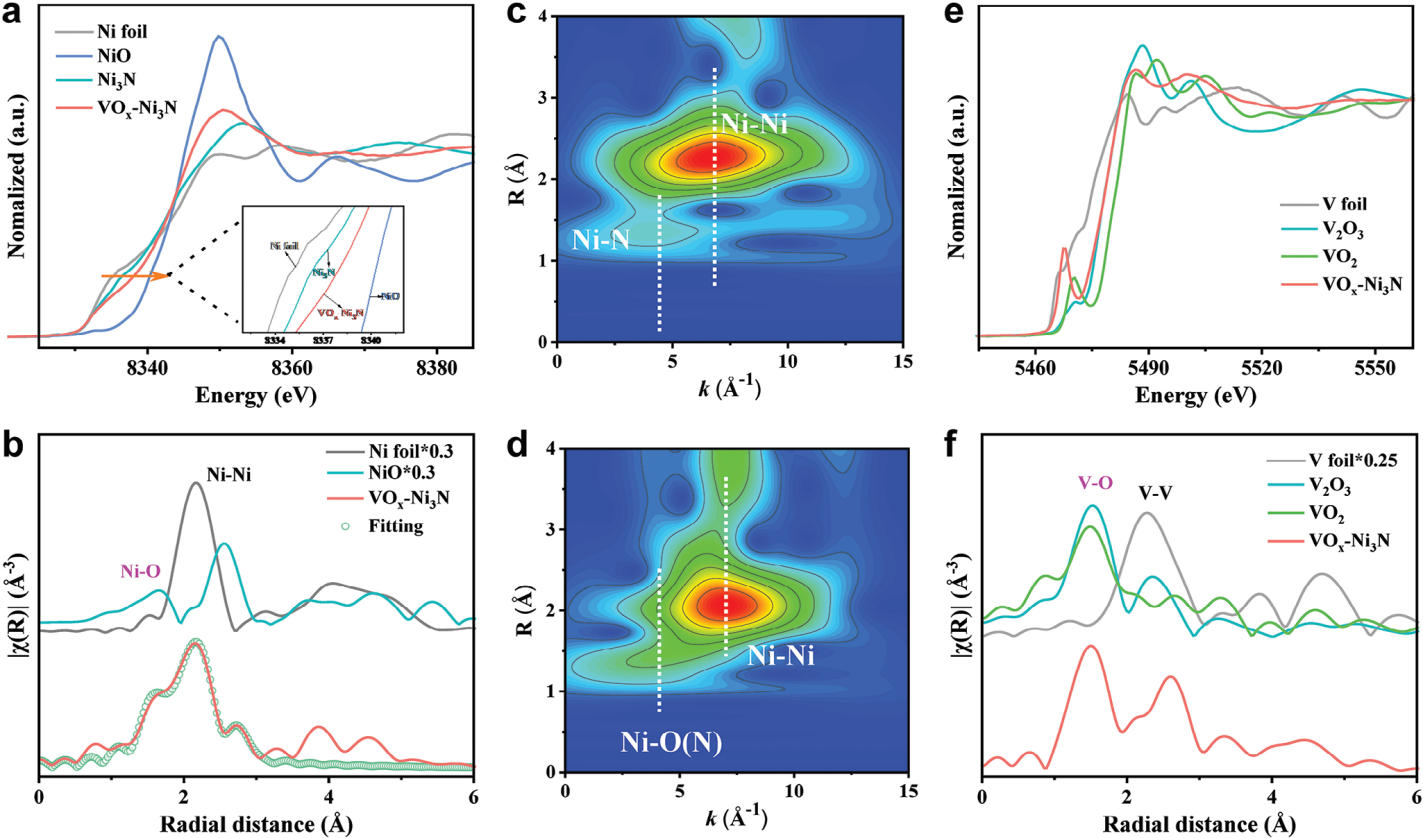
a) Ni K−edge XANES spectra of VO_x_−Ni_3_N, Ni_3_N, NiO, and Ni foil. b) Fourier transforms of the EXAFS (FT−EXAFS) spectra of VO_x_−Ni_3_N, Ni_3_N, NiO, and Ni foil. WT−EXAFS contour plots of the c) Ni_3_N and d) VO_x_−Ni_3_N. e) V K−edge XANES spectra of VO_x_−Ni_3_N, VO_2_, V_2_O_3_ and V foil. f) FT−EXAFS spectra of VO_x_−Ni_3_N, VO_2_, V_2_O_3_ and V foil.

To further clarify the coordination environments and electronic structures of the VO_x_−Ni_3_N heterostructure, specifically the Ni─O─V interface, we performed X‐ray absorption fine structure (XAFS) analyses. As shown in **Figure** **3**a, the X‐ray absorption near edge structure (XANES) spectra of Ni K‐edge indicate that the adsorption edges of both VO_x_−Ni_3_N and Ni_3_N are close to that of the Ni foil. Moreover, VO_x_−Ni_3_N presents a high‐energy shift of the pre‐edge feature, compared with Ni foil and Ni_3_N, suggesting an oxidation of Ni upon forming the Ni─O─V interface.^[^ ^30^, ^31^ ^]^ This is consistent with the binding energy shift in XPS spectra. Figure 3b shows the Fourier transform k^2^‐weighted extended X‐ray absorption fine structure (FT‐EXAFS) spectra (Table S1, Supporting Information). The prominent peak is located at ≈2.16 Å (uncorrected value) for VO_x_−Ni_3_N, assigned to the nitrogen‐bridged Ni─Ni scattering.^[^ ^32^ ^]^ Besides, a scattering peak corresponding to Ni─N/O scattering is located at ≈1.50 Å. Notably, the Ni─N/O scattering increases upon the decrease of the Ni─Ni scattering for VO_x_−Ni_3_N when compared with that of bare Ni_3_N, indicating that the introduced V encourages the formation of the Ni─O─V interface.

The wavelet‐transformed (WT) of Ni K‐edge EXAFS oscillation was employed to illustrate the variation in the coordination structure at high k‐space resolution. Figure 3c,d provide the WT contour spectra of Ni_3_N and VO_x_−Ni_3_N, respectively. They both exhibit intensity maxima at ≈7 Å^−1^, attributed to the prominent Ni─Ni scattering due to the Ni_3_N principle.^[^ ^33^ ^]^ The same intensity maximum also suggests that V stays in a separate phase, rather than insertion into the Ni_3_N framework. For Ni─N/O scattering at 4.4 Å^−1^, the enhanced intensity of Ni─N/O scattering for VO_x_−Ni_3_N further confirms the VO_x_‐mediated Ni─O─V interface. The electronic structure of V in VO_x_−Ni_3_N was probed from the V K‐edge XAFS spectra (Figure 3e). The absorption edge of V in VO_x_−Ni_3_N is comparable with V_2_O_3_. Meanwhile, V mainly coordinates with oxygen in the first scattering shell, consistent with standard vanadium oxides (Figure 3f). The subtle differences observed in the pre‐edge and white‐line intensity imply that the valence state of V in VO_x_−Ni_3_N is somewhat higher than that in V_2_O_3_, not entirely +3. The emerging Ni─O─V interface in VO_x_−Ni_3_N further encourages the charge transfer and results in an increased valence of V. Therefore, the Ni─O─V interface in VO_x_−Ni_3_N is clearly identified, exhibiting prominent charge redistribution accompanied by interfacial polarization.

### 2.2 Electrocatalytic Performance

**Figure 4:**
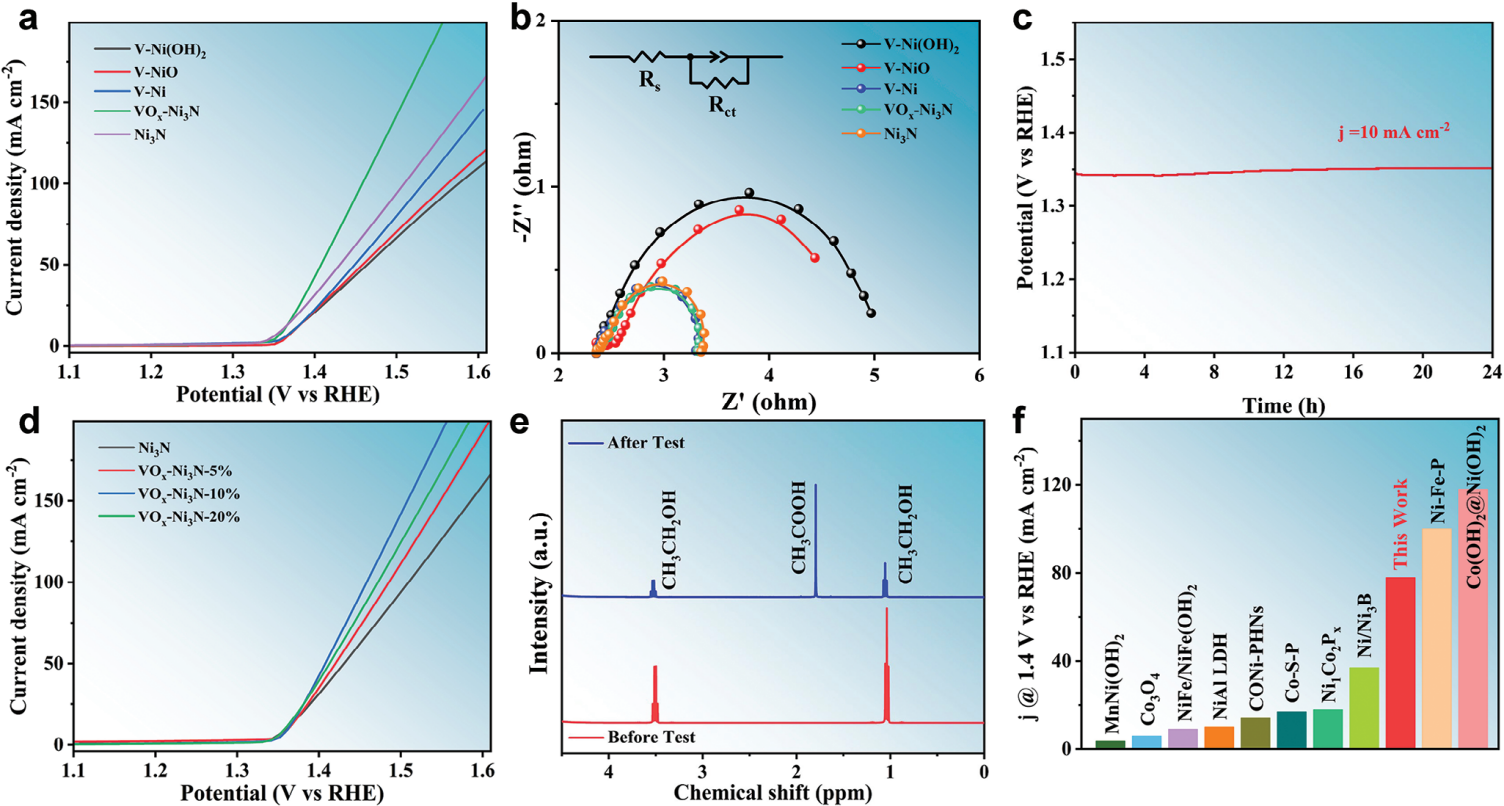
The EOR electrocatalytic properties of the VO_x_−Ni_3_N catalyst in 1 m KOH with 0.25 m EtOH electrolyte (without iR compensation): a) Linear sweep voltammetry (LSV) curves of V−Ni(OH)_2_, V−NiO, V−Ni and VO_x_−Ni_3_N. b) EIS curves. The inset is the equivalent circuit. c) Constant potential V–t curves of VO_x_−Ni_3_N. d) LSV curves of Ni_3_N, VO_x_−Ni_3_N−5%, VO_x_−Ni_3_N−10% and VO_x_−Ni_3_N−20%. e) ^1^H NMR measurements of the product after the reaction. f) Comparison of EOR performance with other transition metal−based catalyst histograms.

The EOR performance of the catalysts was evaluated using a standard three‐electrode system. The test conditions were first optimized by choosing various electrolytes (Figure S3, Supporting Information). The current densities achieved by VO_x_−Ni_3_N after the addition of 0.05, 0.1, 0.25, 0.5, and 1 m ethanol (EtOH)to 1 m KOH were 5, 23, 32, 22, and 14 mA cm^−2^, respectively, at a voltage of 1.4 V versus reversible hydrogen electrode (V_RHE_). Hence, the electrolyte condition was maintained as 1 m KOH with 0.25 M EtOH. Notably, VO_x_−Ni_3_N delivers the desired catalytic activity, outperforming nickel‐based hydroxides, oxides, and nickel metals (**Figure** **4**a). A potential of merely 1.4 V is required to perform a current density of 50 mA cm^−2^, which is far superior than that of V−Ni (1.45 V), V−NiO (1.46), and V−Ni(OH)_2_ (1.47 V). According to the electrochemical impedance spectroscopy (EIS) plots in Figure 4b and Table S2 (Supporting Information), the VO_x_−Ni_3_N shows the smallest charge transfer resistance (R_ct_), indicating the kinetic charge transfer during the EOR process. Also, the durability of the VO_x_−Ni_3_N toward EOR is demonstrated by the steady current density observed at a fixed potential, as depicted in Figure 4c.

The dependence of catalytic performance on the amount of introduced V was also evaluated in Figure 4d and Table S3 (Supporting Information). The findings suggest that incorporating 10% V into VO_x_−Ni_3_N yields the best catalytic performance. Meanwhile, VO_x_−Ni_3_N−10% (with 10% vanadium content) exhibits the lowest Tafel slope and charge transfer resistance (R_ct_), as depicted in Figure S4 (Supporting Information). This suggests that the specific incorporation of vanadium significantly facilitates the electron transfer rate, which in turn optimizes the electrocatalytic activity for EOR. Besides, the electrochemically active surface area (ECSA) of the catalysts is measured to further evaluate the effect of surface area on the electrochemical performance (Figure S5, Supporting Information). Upon the incorporation of V, the ECSA exhibits a slight reduction compared to the pristine Ni_3_N. According to the ECSA normalized catalytic activity, VO_x_−Ni_3_N−10% behaves the optimal EOR performance, consistent with the result in Figure 4d. This result indicates that the ECSA is not the primary factor responsible for the observed variations in catalytic performance. Moreover, the lower VO_x_ component will reduce the interface component, potentially hindering the catalytic process. Conversely, an elevated VO_x_ content may lead to the encapsulation of Ni_3_N by amorphous VO_x_, which could compromise the availability of active nickel sites. Consequently, an intermediate loading content of ≈10% of vanadium is proposed to strike a balance between maintaining dual‐polarization and ensuring the exposure of the nickel species necessary for optimal catalytic activity. Thus, the enhanced catalytic activity of VO_x_−Ni_3_N is mainly attributed to the intrinsic activity of the embedded Ni–O–V interface within the VO_x_−Ni_3_N.

As shown in Figure 4e, the products before and after the reaction were evaluated by using ^1^H NMR spectroscopy. The chemical shifts ≈1.0 and 3.5 ppm before EOR correspond to the triple and quadruple peaks of the hydrogen proton in ethanol, respectively.^[^ ^18^ ^]^ After EOR, the intensity of the two peaks decreases appreciably, indicating that most of the ethanol was consumed. Additionally, a new peak appears at 1.7 ppm, which is attributable to the methyl group adjacent to the carboxylate in potassium acetate.^[^ ^34^ ^]^ Therefore, the EOR process follows a 4e^−^ reaction pathway: ^*^CH_3_CH_2_OH→^*^CH_3_CH_2_O →^*^CH_3_CHO→^*^CH_3_CO→^*^CH_3_COOH. This catalytic performance of VO_x_−Ni_3_N toward EOR is benchmarked against the state‐of‐the‐art nickel‐ and cobalt‐based catalysts (Figure 4f; Table S4, Supporting Information).

### 2.3 Catalytic Mechanism

**Figure 5:**
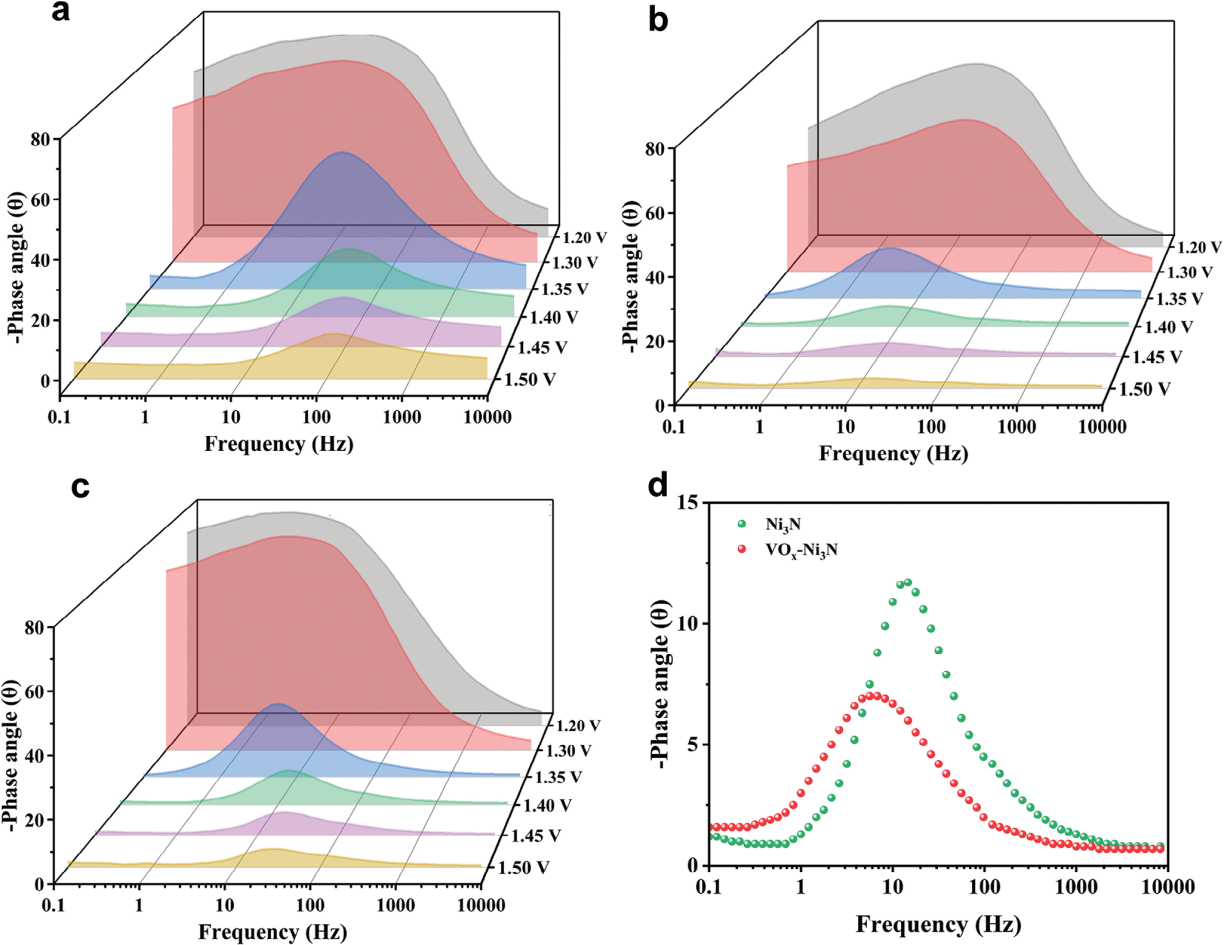
The operand electrochemical impedance spectroscopy characterization: Bode plots of a) V─Ni(OH)_2_, b) VO_x_−Ni_3_N, and c) Ni_3_N electrode for EOR in different potentials. d) Bode phase plots of VO_x_−Ni_3_N and Ni_3_N at 1.4 V _RHE_.

To gain an insight into the catalytic mechanism of the Ni─O─V interface on the EOR process over VO_x_−Ni_3_N, Operando electrochemical impedance spectroscopy was employed to investigate the potential‐dependent interfacial behavior.^[^ ^35^, ^36^ ^]^ **Figure** **5**a,b describe the Bode plots of V─Ni(OH)_2_ and VO_x_−Ni_3_N. Before 1.35 V, there is only a high‐frequency interface with a very weak charge transfer.^[^ ^37^ ^]^ After 1.35 V, there is a sudden decrease in the phase angle, suggesting that the catalyst initiates its reaction. The interfacial response of V─Ni(OH)_2_ in the high‐frequency region indicates that the dominant reaction process is the dehydrogenation of Ni(OH)_2_ species into Ni(OH)O species. In contrast, the interfacial response of VO_x_−Ni_3_N occurs in the low‐frequency region, indicating that the reaction occurs at the catalyst surface, involving adsorbed species such as OH^*^ or O^*^.

Bode plots were utilized for various ethanol concentrations to determine the primary adsorbed species on the catalyst. As illustrated in Figure S6 (Supporting Information), the pre‐oxidation reactions, specifically the adsorption of OH^−^, are consistently maintained between 1.2 to 1.3 V, even with the escalation of ethanol concentrations. Notably, there is no discernible reduction in potential below 1.35 V. This observation points to the low reactivity of OH^*^ adsorption during EOR, thereby suggesting that the dominant active species in the process is more likely to be O^*^.^[^ ^38^ ^]^ Besides, at low ethanol concentrations, the generated O^*^ species are not immediately utilized. Whereafter, an accumulation of O^*^ occurs, resulting in the formation of an oxide layer on the catalyst's surface as the potential rises. As a result, OER begins competing with the EOR, consequently diminishing the selectivity of the EOR. According to Figure S6c–d (Supporting Information), the phase angle frequency exhibits a positive shift with an increase in ethanol concentration, suggesting that an excess of ethanol impedes the adsorption of active species. This interference with adsorption is suggested to be the cause of the observed decline in EOR activity. The variation in phase angle values between the phase peaks of 1.2 and 1.3 V represents the strength of OH^−^ adsorption before the EOR. In comparison to Ni_3_N (Figure 5c), the greater phase angle difference observed for VO_x_−Ni_3_N suggests more robust adsorption of OH^−^. The phase angles of the two samples were also further compared at a voltage of 1.4 V_RHE_. It is found that the phase peaks of VO_x_−Ni_3_N at the low‐frequency interface show smaller phase angles and displacements (Figure 5d), This observation suggests a kinetic deprotonation process of the oxygen‐containing intermediate OH^*^ during the EOR process over VO_x_−Ni_3_N.^[^ ^39^, ^40^ ^]^

**Figure 6:**
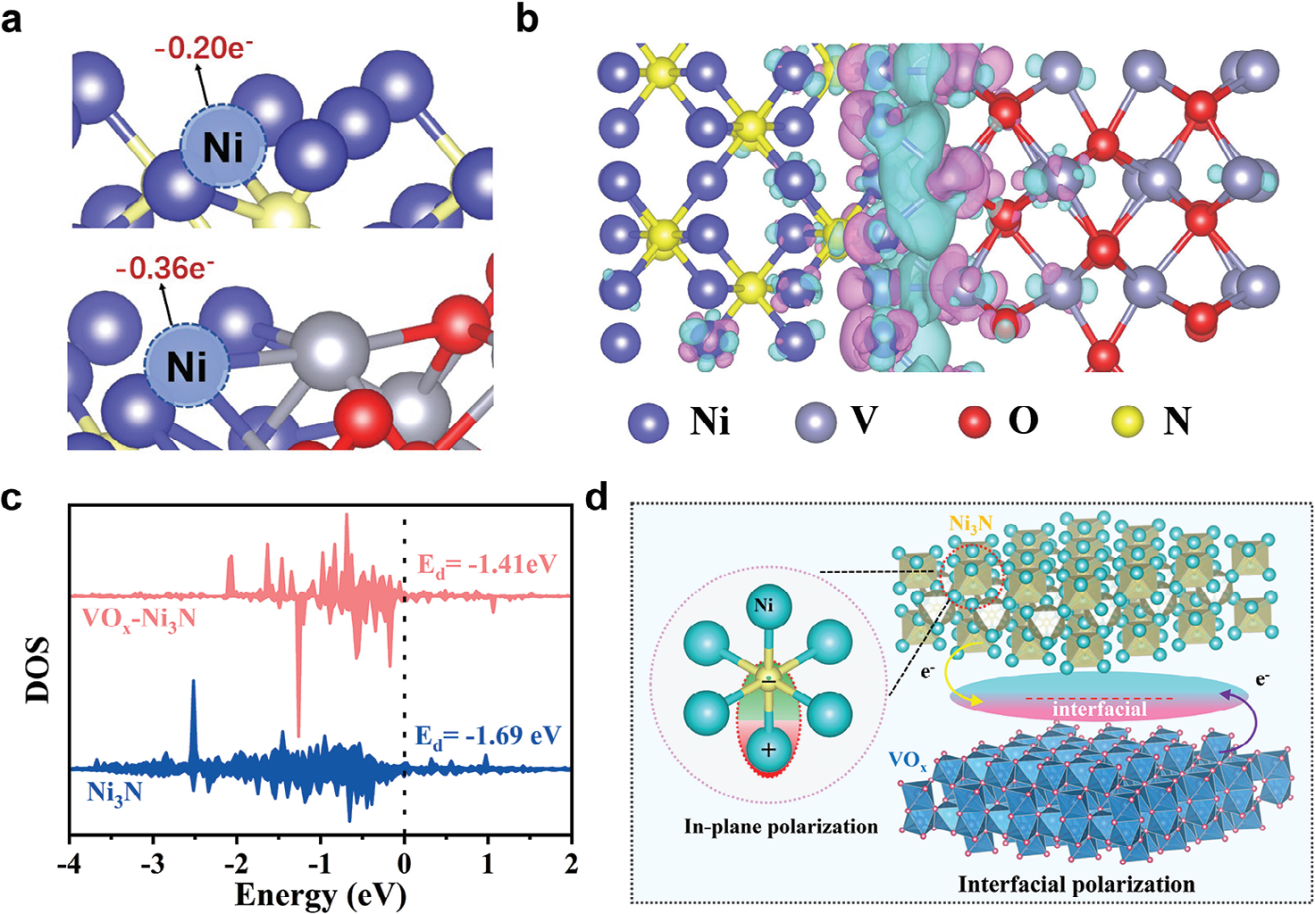
DFT calculation results of the HER process on the catalyst surfaces: a) Differential charge density of VO_x_−Ni_3_N heterostructure. Cyan and pink indicate charge accumulation and consumption, respectively. b) Bader charge analysis of Ni_3_N and VO_x_−Ni_3_N. c) Density of states (DOS) of Ni−d orbitals in Ni_3_N and VO_x_−Ni_3_N. d) Schematic of dual polarization in VO_x_−Ni_3_N.

Density Functional Theory (DFT) calculations have provided insights into the catalytic activity for EOR over the Ni─O─V interface. It is well understood that heterogeneous components possess distinct work functions, which naturally give rise to an internal electric field (IEF).^[^ ^41^ ^]^ The charge difference density distribution, as depicted in **Figure** **6**a, indicates that the built‐in IEF encourages a pronounced charge redistribution at the Ni─O─V interface. The delocalized electron further promotes the polarization of the Ni─O─V interface, thereby facilitating the transfer of electrons at the interface.^[^ ^42^ ^]^ Figure 6b provides the calculation of the Bader charge value for the Ni site on pristine Ni_3_N and VO_x_−Ni_3_N. The average Bader charge value of Ni on Ni_3_N was calculated to be −0.20 e, signifying electrons transfer from Ni to N. This electron transfer suggests the occurrence of spontaneous in‐plane lattice polarization, where Ni atoms lose electrons into positively charged, and N atoms gain electrons into negatively charged. In the case of the Ni─O─V interface in VO_x_−Ni_3_N, the Bader charge value of −0.36 e for Ni indicates a significant electron transfer from Ni_3_N to VO_x_. This interfacial polarization is instrumental in leading to an increased valence state of Ni at the Ni─O─V interface. This charge transfer is in alignment with the electronegativity order: O (3.44) > N(3.04) > Ni (1.91) > V (1.63). As a result, the dual polarization at the Ni─O─V interface, particularly in‐plane polarization, and IEF−induced interfacial polarization, plays a crucial role in modulating the electronic properties of the Ni site, especially the increase in the d‐band center of Ni.

According to the d‐band center (ε_d_) theory, the lower energy level of the d‐band center indicates a higher occupancy of the antibonding states, which correlates with weaker adsorption toward intermediates.^[^ ^43^ ^]^ As the density of state depicted in Figure 6c, the ε_d_ value of VO_x_−Ni_3_N is −1.41 eV, higher than that of pristine Ni_3_N (−1.69 eV). As illustrated in Figure 6d, the dual polarization at the Ni─O─V interface could effectively optimize the adsorption of key intermediates, such as OH^−^, and kinetically promote the formation of reactive species O^*^.^[^ ^44^ ^]^

**Figure 7:**
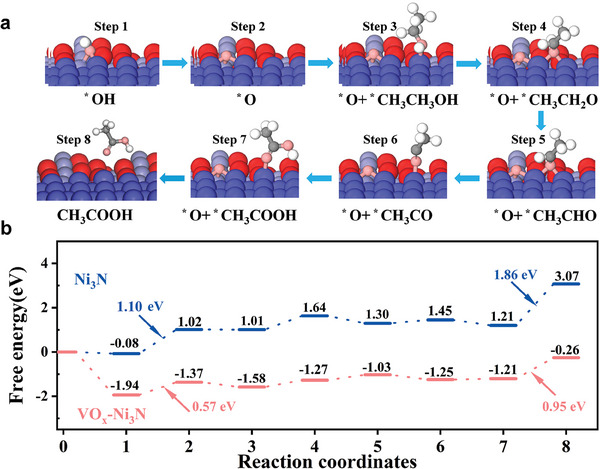
DFT calculation for the reaction of ethanol on Ni_3_N and VO_x_−Ni_3_N. a) The reaction coordinates (a) and the corresponding Gibbs free energies b) during the EOR process. The blue, cyan, red, grey, and white balls represent nickel, vanadium, oxygen, carbon, and hydrogen, respectively.

Furthermore, we calculated the Gibbs free energy required for the EOR process (**Figure** **7**). For the key step of the formation of reactive O^*^, an uphill energy of 1.1 eV is required for Ni_3_N, while this energy drops to 0.57 eV for Ni─O─V interface‐mediated VO_x_−Ni_3_N. The calculated result is quite consistent with the in situ electrochemical impedance analyses. Moreover, the Ni sites in the Ni─O─V interface were tuned for favorable adsorption for all involved intermediates. A more energetic desorption (0.95 eV) of acetic acid over the Ni─O─V interface is demonstrated in comparison to pristine Ni_3_N (1.86 eV). Therefore, DFT calculation clarifies the dual polarization within the Ni─O─V interface in VO_x_−Ni_3_N heterostructure, which substantially encourages the EOR process thermodynamically.

### 2.4 Hybrid Electrolyzer

**Figure 8:**
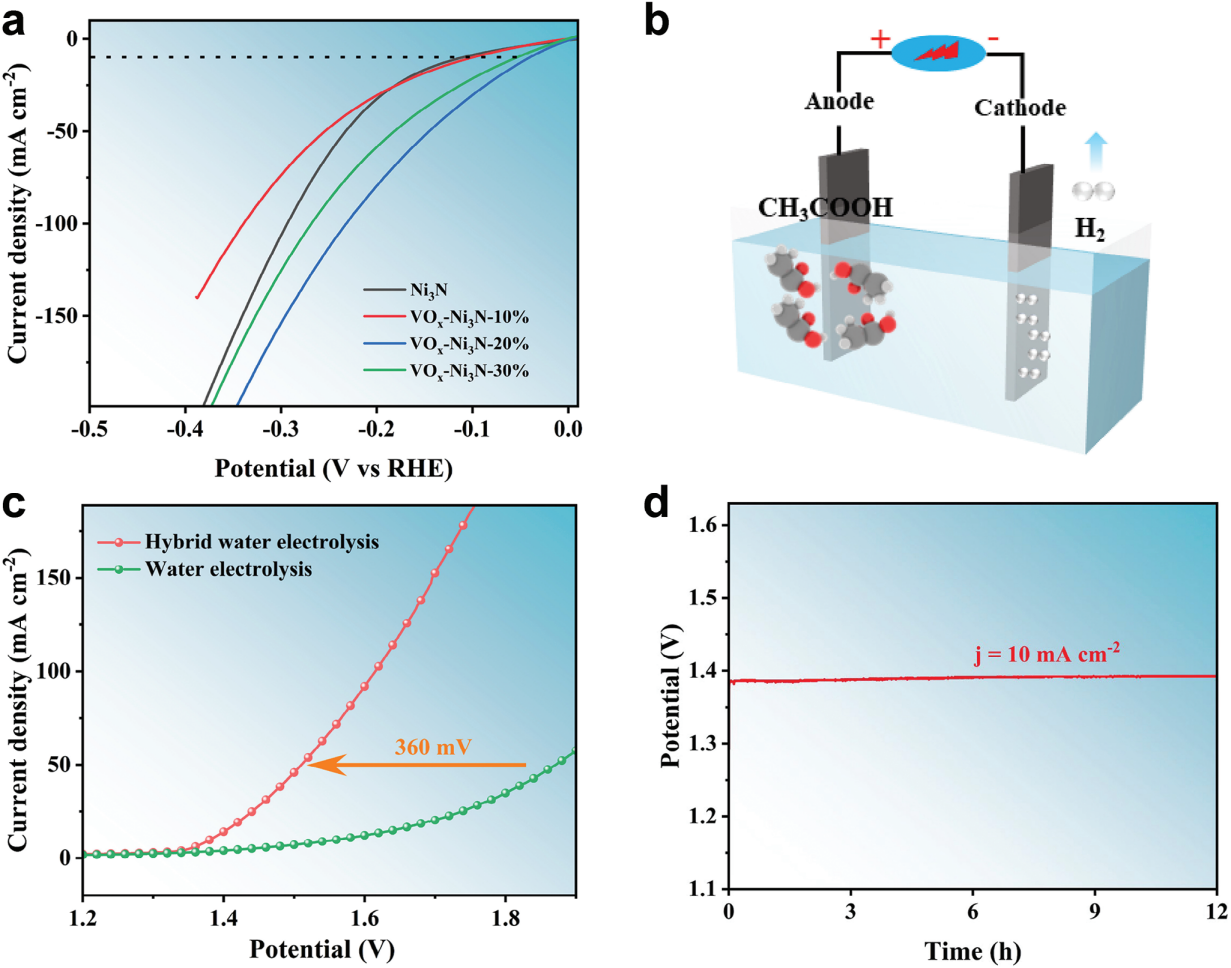
HER performance in 1 m KOH + 0.25 m EtOH electrolyte (without iR compensation): a) LSV curves of Ni_3_N, VO_x_−Ni_3_N−10%, VO_x_−Ni_3_N−20% and VO_x_−Ni_3_N−30%. b) schematic diagram of the HER coupled EOR hybrid electrolysis. c) LSV curves of the hybrid and conventional hydrolysis, where VO_x_−Ni_3_N−10% was used as the anode and VO_x_−Ni_3_N−20% as the cathode. d) V−t diagrams of the hybrid hydrolysis.

In a further study, it was revealed that a proper Ni─O─V interface could also significantly enhance the HER catalytic activity. As shown in **Figure** **8**a, VO_x_−Ni_3_N exhibits an excellent HER catalytic performance in alkaline conditions. Specifically, when the proportion of vanadium is 20% (VO_x_−Ni_3_N−20%), the required overpotential is 60.3 mV. This value is significantly lower than that of Ni_3_N (117.6 mV), VO_x_−Ni_3_N−10% (101.4 mV), and VO_x_−Ni_3_N−30% (81.7 mV). The superior hydrogen evolution reaction (HER) performance exhibited by VO_x_−Ni_3_N−20% as opposed to VO_x_−Ni_3_N−10% can likely be attributed to the increased presence of VO_x_‐induced Ni–O species, which are essential for water dissociation in the alkaline HER process The corresponding Tafel and impedance analyses (Figure S7a−b, Supporting Information) signify that the dual polarization within the Ni─O─V interface could synergistically promote the Volmer step and the sequential hydrogen production.^[^ ^45^ ^]^ Besides, the desired durability of VO_x_−Ni_3_N for alkaline HER is demonstrated as 24 h of steady catalysis at 10 mA cm^−2^ (Figure S7c, Supporting Information).

Due to the outstanding bifunctional application of VO_x_−Ni_3_N for both EOR and HER, a hybrid electrochemical cell was established (Figure 8b). The anodic chamber contains KOH and ethanol at the concentrations of 1 and 0.25 m, respectively; while the cathodic chamber only contains KOH at the concentration of 1 m. Figure 8c,d demonstrate that the system offers outstanding catalytic performance and favorable durability in ethanol oxidation coupled hydrogen production. At a current density of 50 mA cm^−2^, a cell voltage of ≈1.5 V is required, which is 300 mV lower than overall water splitting. Meanwhile, the performance is significantly superior to many mixed electrolytic water catalyst systems with non−precious metals (Tables S5−S6, Supporting Information). Therefore, creating dual‐polarization by precisely engineering an interface within heterostructure is of great significance toward broad catalysis.

## 3 Conclusion

In summary, we have meticulously crafted a heterostructure that integrates amorphous vanadium oxide with crystalline nickel nitride (VO_x_−Ni_3_N), featuring a well‐defined Ni─O─V interface. The catalyst demonstrates high‐efficiency ethanol oxidation into acetic acid, specifically delivering a current density of 50 mA cm^−2^ at 1.38 V_RHE_, significantly surpassing pristine Ni_3_N. Various characterization techniques and theoretical calculations elucidate the dual polarization, particularly the interfacial polarization induced by the internal electric field and the in‐plane polarization within the Ni─O─V interface, which encourages the modification of electronic structure, thereby facilitating the EOR process. Given the superior catalytic performance of VO_x_−Ni_3_N toward alkaline hydrogen evolution, an EOR‐coupled‐HER electrolyzer was established. A commercial dry battery (1.5 V) is capable of driving the electrolyzer to deliver a current density of 50 mA cm^−2^, far surpassing the traditional overall water−splitting electrolyzer both in energy conservation and the production of value‐added products. This strategy of precisely engineering interface in heterostructure is applicable to a broad range of applications.

## Conflict of Interest

There is no conflict of interest to declare.

## Author Contributions

M.Z., B.J., W.K., and Y.C. performed the experimental investigation. A.C. and X.Z. performed the theoretical investigations. M.Z. wrote the manuscript. F.L. and X.Z. revised the manuscript. F.L., X. W., and X.Z. supervised this work.

## Supporting information

Supporting Information

## Data Availability

The data that support the findings of this study are available from the corresponding author upon reasonable request.
