# Supplementary material for: Dual Polarization of Ni Sites at VOx−Ni3N Interface Boosts Ethanol Oxidation Reaction
Source: Adv Sci (Weinh). 2024 Sep 3;11(40):2407473. doi: 10.1002/advs.202407473 (PMC11516135; doi:10.1002/advs.202407473)

Supporting Information

Dual Polarization of Ni Sites at VO_x_−Ni_3_N Interface Boosts Ethanol Oxidation Reaction

Min Zhou, Binrong Jin, Weijie Kong, Anjie Chen, Yuhe Chen, Xiuyun Zhang, Fei Lu*, Xi Wang*, Xianghua Zeng*

**Experimental methods**

1. Experimental reagents

Nickel nitrate hexahydrate, vanadium chloride, urea, ammonium fluoride, potassium hydroxide, hydrochloric acid and acetone were purchased from Sinopharm Chemical Reagent Co. Ltd and Aladdin without any impurity. All the reagents were analytically pure. Millipore water (resistivity: ∼18 MΩ·cm) was used in all experiments.

2. Synthesis of VO_x_−Ni_3_N

VO_x_−Ni_3_N was synthesized via two steps. Specifically, 1.35 mmol of Ni(NO_3_)_2_·6H_2_O and 0.15 mmol of VCl_3_ were dissolved in 30 mL of deionized water (DI) under full stirring, followed by the addition of 7.5 mmol urea and 3 mmol ammonium fluoride. Stirring for 15 min, then treated NF was immersed in it and reacted at 100 °C for 4 h. After cooling down to room temperature, the precursor was washed with deionized water and ethanol several times and dried at 60℃ for 4 h.

The VO_x_−Ni_3_N heterointerface can be fabricated by high−temperature vapor deposition. The specific experimental procedure is as follows: the precursor is put into a tube furnace and reacted under NH_3_ atmosphere at 400 °C for 2 h. The heating rate is 10 °C/min. The other comparison samples were prepared under uniform conditions, except for the different atmospheres required for annealing; V−Ni was synthesized under an atmosphere of H_2_/Ar (95%) and V−NiO was prepared in air.

3. Materials characterizations

The phase structures of samples were identified by Shimadzu X−ray diffraction (XRD)−7000 powder x−ray diffractometer (Cu Ka). The morphologies and structures of the products were characterized by Tecnai G2 F30 S−TWIN transmission electron microscopy (TEM). X−ray photoelectron spectroscopy (XPS) measurements were performed on a Thermo ESCALAB250Xi spectrometer with a resolution of 0.43 eV. X−ray absorption fine structure (XAS) was characterized in fluorescence mode at beamline 1W1B at Beijing Synchrotron Radiation Facility (BSRF) and beamline BL14W1 at Shanghai Synchrotron Radiation Facility (SSRF). The products were analyzed on a liquid Nuclear Magnetic Resonance (NMR) spectrometer Bruker AVANCE III HD−400MH from Bruker, Germany.

4. Electrochemical measurements

The electrocatalytic activity was evaluated in a three−electrode system in 1 M KOH with 0.25M EtOH as electrolyte using a CHI 760E electrochemistry workstation (Chenhua, Shanghai). Linear sweep voltammetry (LSV) was performed at a scan rate of 5 mV/s. Electrochemical impedance spectroscopy (EIS) was performed at a cathode bias of 0.4 V vs RHE, using a sinusoidal voltage of 5 mV in the range of 10 kHz−0.1 Hz. At 0.35−0.45 V vs RHE region, a series of CV measurements were performed at different sweep rates (10−100 mV s^−1^). Stability was tested at 10 mA cm^−2^ for constant current V−t curves.

5. Density functional theory (DFT) calculations

All DFT calculations were performed in the Vienna ab initio simulation package (VASP) using projector−augmented wave (PAW) potentials. The exchange−correlation energy is described by the generalized gradient approximation (GGA) with Perdew− Burke−Ernzerholf (PBE) functional. The cutoff value of the plane wave basis set is set to 450 eV. We employed a slab model where the vacuum spacing perpendicular to the surface layer is larger than 26 Å to eliminate physical interactions due to periodic boundary conditions along this direction. The convergence threshold of the force is reached when the Hellmann−Feynman force on each atom is less than 0.01 eV/Å. 1 × 1 × 1 and 3× 3 × 1 Γ−centered k−grids are used for geometric optimization and electronic structure calculations.


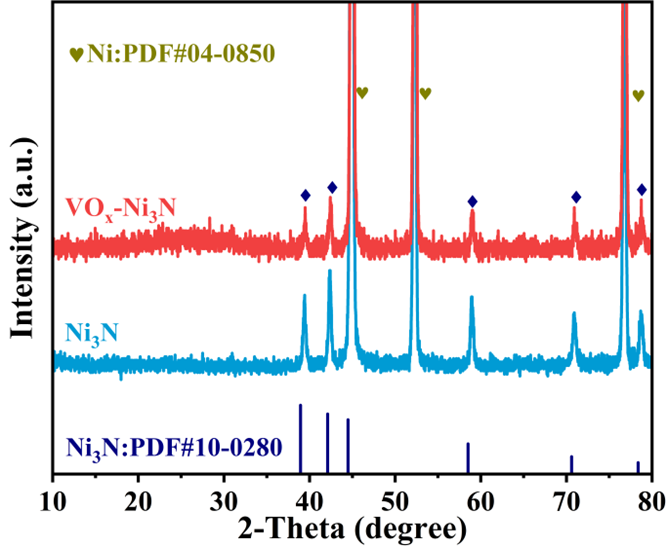


**Figure S1.** XRD pattern of the Ni_3_N and VOx−Ni_3_N.


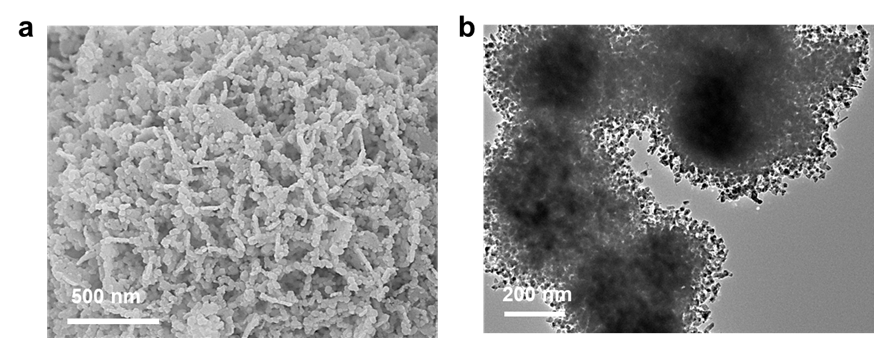


**Figure S2.** (a) SEM images of VO_x_−Ni_3_N, (b) TEM image.


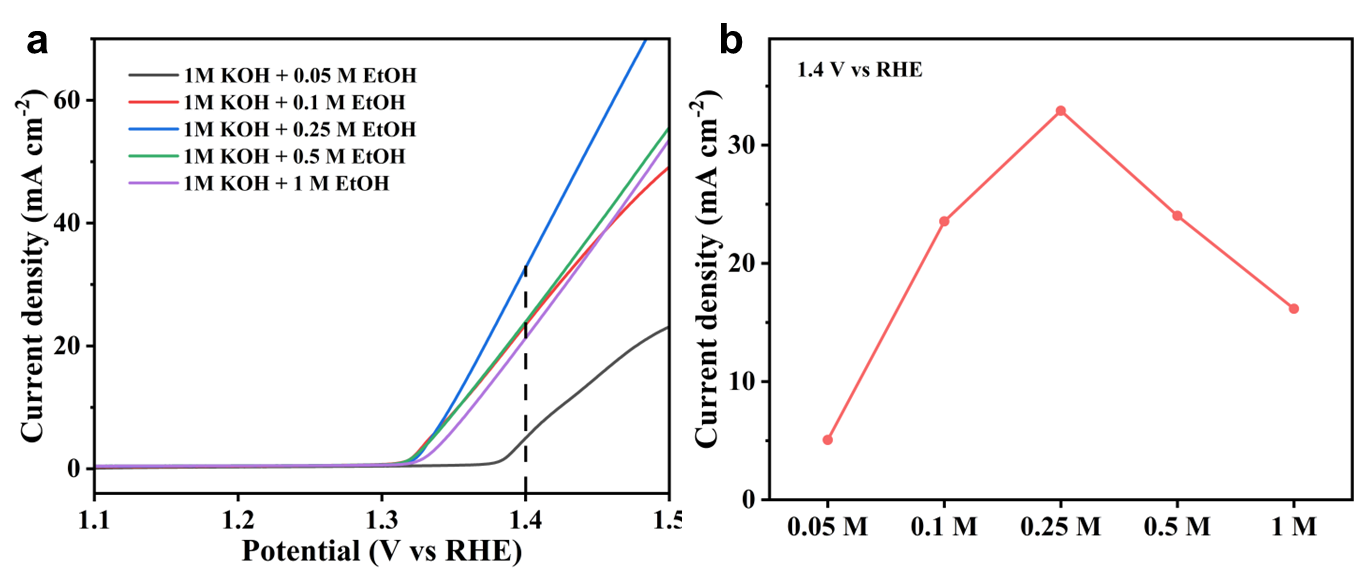


**Figure S3.** (a) LSV curves of different contents of ethanol, the catalyst was a powder sonicated off the substrate. (b) Potential At 1.4 V_EHE_ comparison histogram.


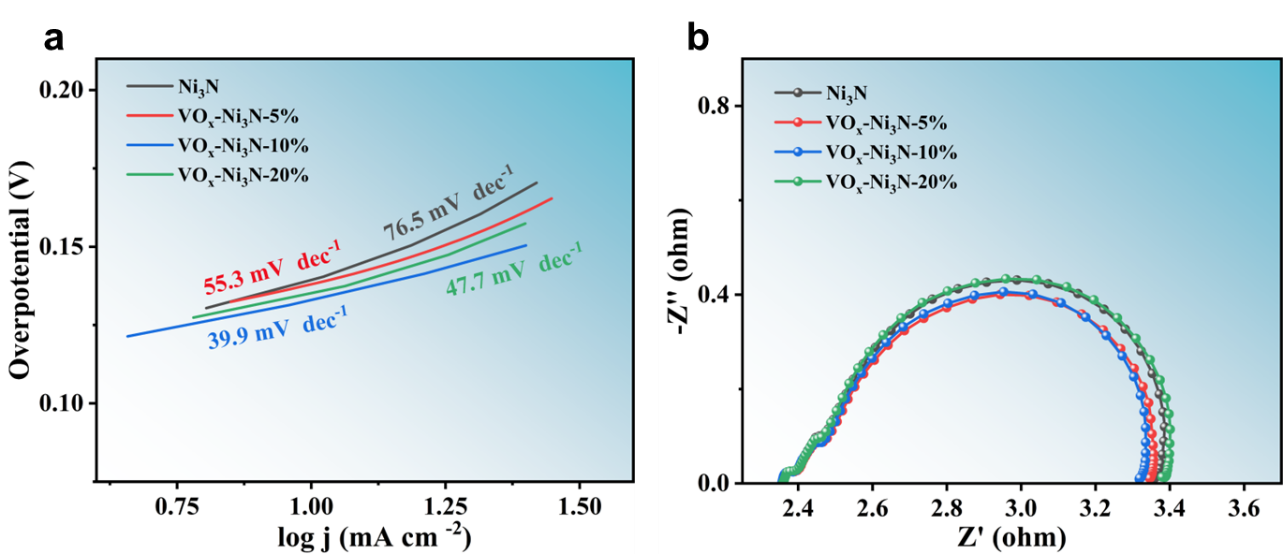


**Figure S4.** (a) Tafel slope plots of different contents of V. (b) Electrochemical impedance (EIS).

**Figure S5.** CV curves of (a) Ni_3_N. (b) VO_x_−Ni_3_N−5%. (c) VO_x_−Ni_3_N−10%. (d) VO_x_−Ni_3_N−20. (e) Current density versus scan rates in 1 M KOH solution. (f) The ECSA normalized catalytic performance of VO_x_−Ni_3_N at different VO_x_ contents.


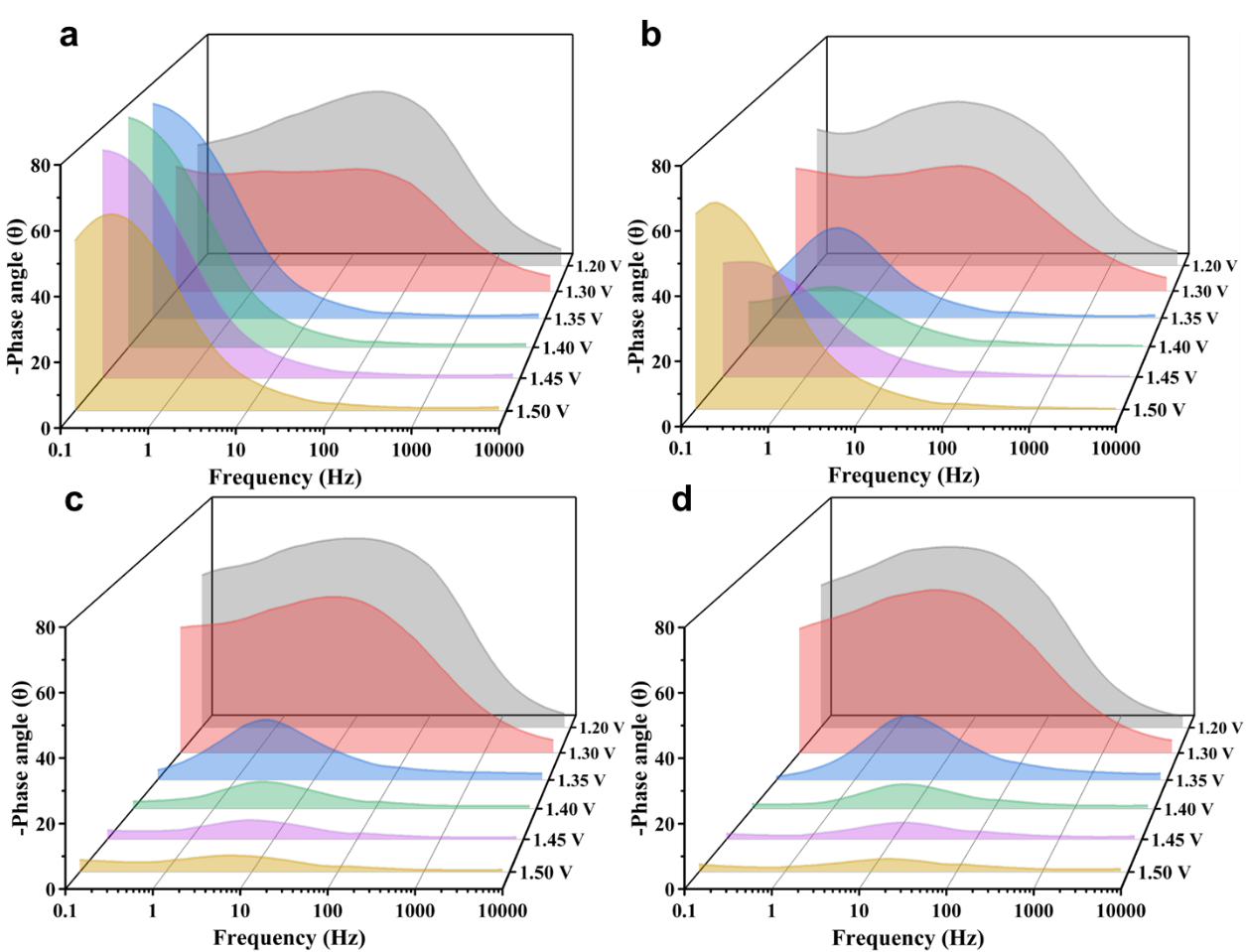


**Figure S6.** Bode plots of VO_x_−Ni_3_N (a) 1 M KOH. (b) 1 M KOH +0.05 M Ethanol. (c) 1 M KOH +0.25 M Ethanol. (d) 1 M KOH +1 M Ethanol.


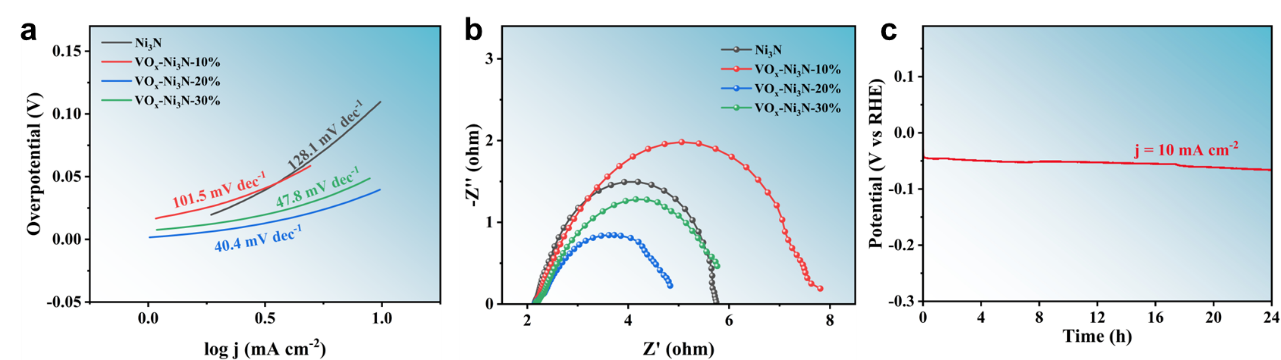


**Figure S7.** **(a)** Tafel slope plots of Ni_3_N, VO_x_−Ni_3_N−10%, VO_x_−Ni_3_N−20% and VO_x_−Ni_3_N−30%. (b) EIS plots. (c) V-t plot of constant potential for VO_x_−Ni_3_N−20% catalyst.

**Table S1.** Fitting results for Ni_3_N and VO_x_−Ni_3_N.
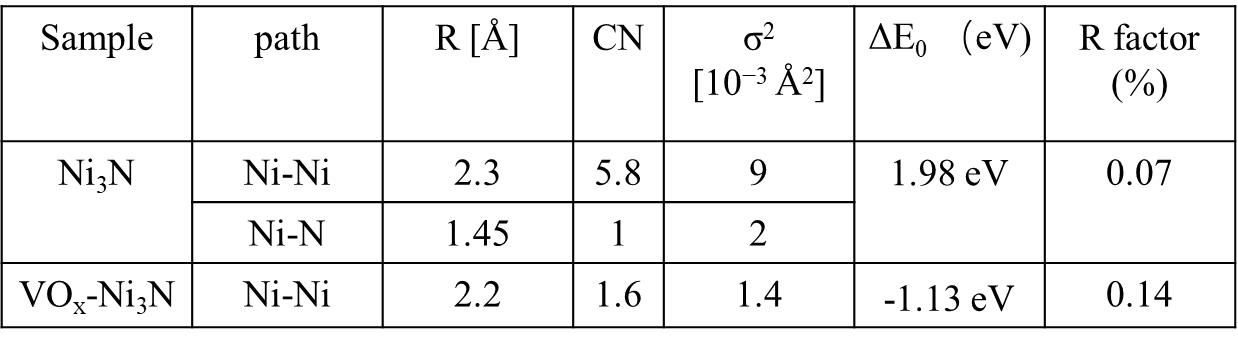


**Table S2.** The key parameter, charge transfer resistance, extracted from the fitting of the Nyquist plot.

| Samples | V-Ni(OH)_2_ | V-NiO | V-Ni | VO_x_-Ni_3_N | Ni_3_N |
| --- | --- | --- | --- | --- | --- |
| R_ct_ | 2.70 | 2.60 | 0.98 | 0.95 | 1.00 |

**Table S3.** The ratios in weight percentage of V and Ni extracted from the ICP-OES characterization.

| sample | Ni wt.% | V wt.% |
| --- | --- | --- |
| VO_x_−Ni_3_N–10% | 83.2 | 7.6 |
| VO_x_−Ni_3_N–20% | 67.6 | 15.7 |
| VO_x_−Ni_3_N–30% | 56.8 | 23.2 |

**Table S4.** Comparisons of the EOR performance of VO_x_−Ni_3_N and other electrocatalysts.


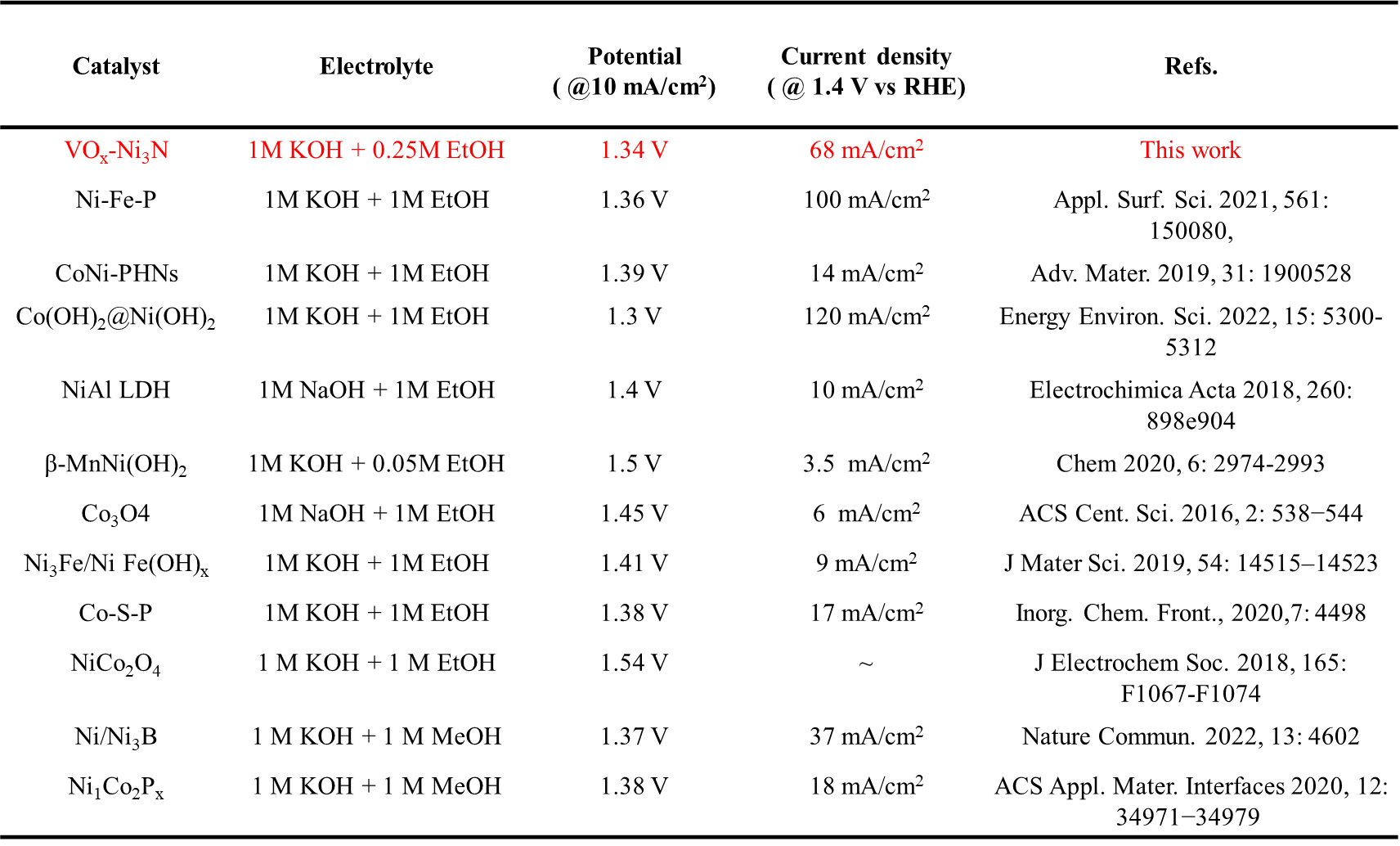


**Table S5.** Comparisons of the HER coupled EOR performance of VO_x_−Ni_3_N and other electrocatalysts.
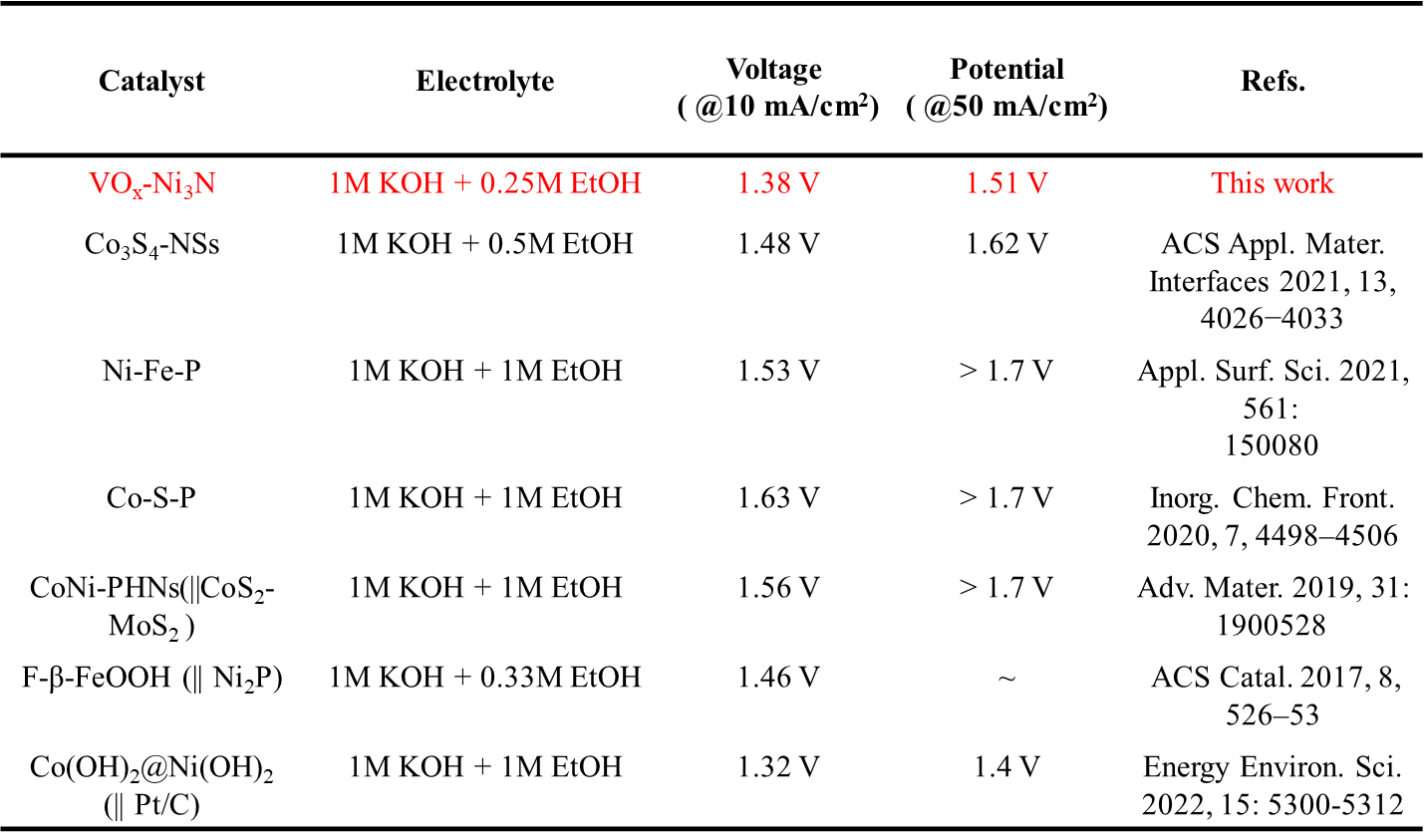


**Table S6.** Comparisons of the HER coupled EOR performance of VO_x_−Ni_3_N and other electrocatalysts for the HER coupled to other organic oxidation reactions.
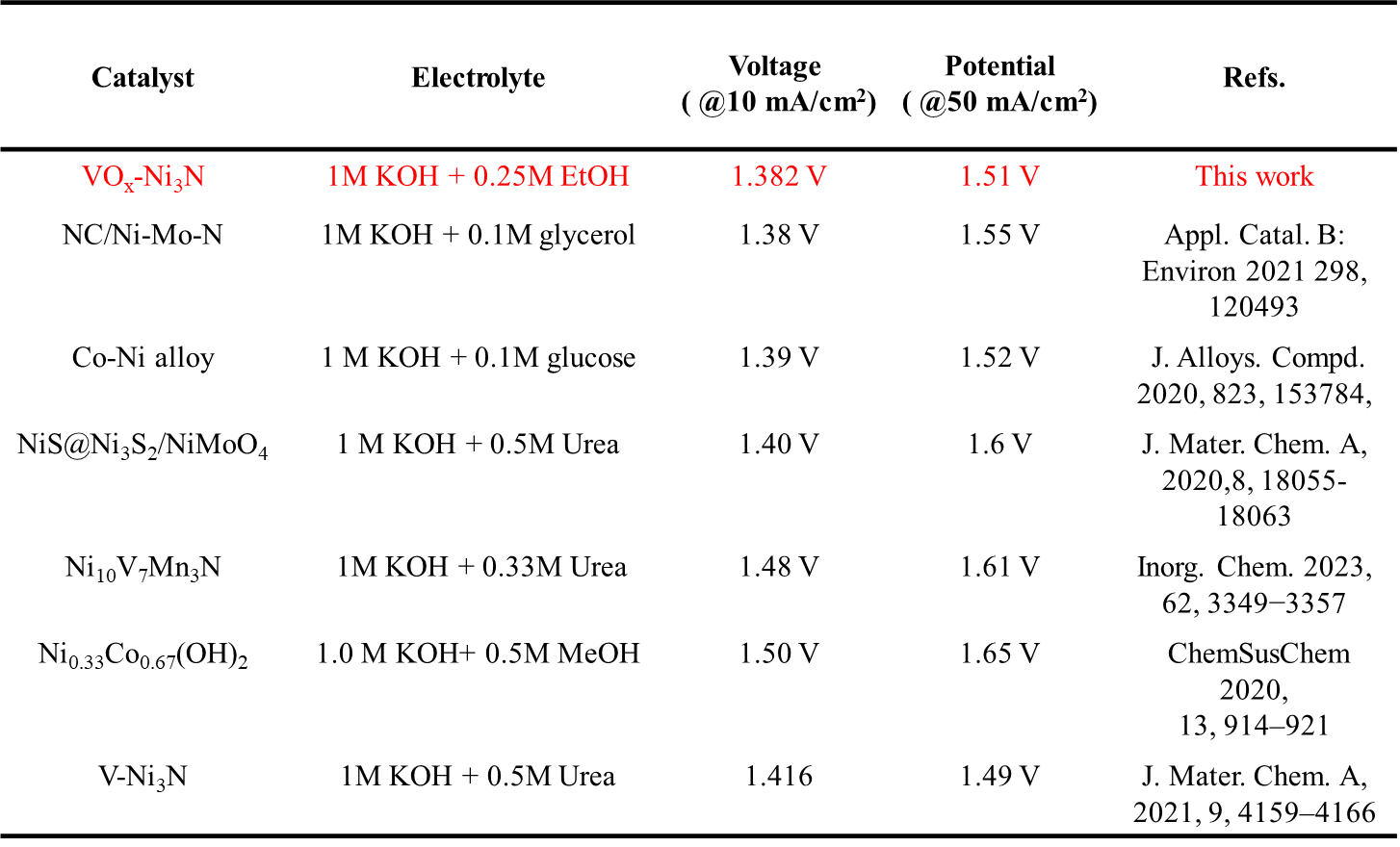

Supplement: Supplementary file 1 — Supporting Information [file ADVS-11-2407473-s001.docx]
